# The Effect of a Novel Silver-Citrate Root Canal Irrigation Solution (BioAkt), Ethylenediamine Tetraacetic Acid (EDTA), and Citric Acid on the Microhardness of Root Canal Dentin: A Comparative In Vitro Study

**DOI:** 10.7759/cureus.31255

**Published:** 2022-11-08

**Authors:** Anas A Alyahya, Mohamad Salem Rekab, Alaa Eddin O AL-Ostwani, Anas Abdo, Kamal Kayed

**Affiliations:** 1 Department of Endodontics, Damascus University, Damascus, SYR; 2 Department of Pediatric Dentistry, International University for Science and Technology, Damascus, SYR; 3 Department of Pediatric Dentistry, Madina Dental Centre, Doha, QAT; 4 Department of Physics, Damascus University, Damascus, SYR

**Keywords:** chelating agents, dentin microhardness, citric acid, bioakt, edta

## Abstract

Background and objective

The use of a chelating agent as an adjunct to disinfectant irrigation is very necessary to remove the smear layer formed during root canal treatment. However, the decalcifying solutions have a negative impact on dentin microhardness, which might affect the result of endodontic therapy. The aim of this study is to evaluate the effect of 17% ethylenediamine tetraacetic acid (EDTA), a novel silver-citrate root canal irrigation solution (BioAkt), 10% citric acid, and 40% citric acid on the microhardness of root canal dentin.

Materials and methods

Forty-five single-root teeth were distributed equally into five groups A, B, C, D, and E treated with distilled water as a control, 17% EDTA, BioAkt, 10% citric acid, and 40% citric acid, respectively. The dentin microhardness was measured before and after the experiment, and the collected data were analyzed using paired sample T-test, One-way ANOVA test, and least significant difference (LSD) test (multiple comparisons), (P < 0.05).

Result

The results showed that the averages of dentin microhardness values before the experiment were 62.83 kg/mm², 65.34 kg/mm², 64.79 kg/mm², 62.95 kg/mm², and 56.47 kg/mm² for groups A, B, C, D, and E, respectively, while the averages after the experiment were 62.60 kg/mm², 54.92 kg/mm², 54.50 kg/mm², 51.31 kg/mm², and 49.37kg/mm² accordingly. Statistical analysis revealed that 17% EDTA, BioAkt, 10% citric acid, and 40% citric acid decreased the dentin microhardness significantly comparing with the negative control group, without statistical differences among them.

Conclusion

The chelating agents 17% EDTA, BioAkt, 10% citric acid, and 40% citric acid declined the microhardness of root canal dentin similarly, which might have possible effects on endodontic treatment and the properties of root canal filling materials as well.

## Introduction

Historically, a wide variety of endodontic irrigants have been used to remove dentin debris and disinfect the root canals as well [1]. The most common is sodium hypochlorite (NaOCL) which has the capability to dissolve organic remaining tissue in addition to its antibacterial and antifungal properties. On the other hand, NaOCL has limited ability to remove the smear layer, which is formed during root canal preparation and consists of microorganisms, pulp tissue debris, inorganic particles, and denatured dentin, thus necessitating the combination use with chelating agents [2]. Ethylenediamine tetraacetic acid (EDTA), phosphoric acid, citric acid, maleic acid, tetracycline isomer with acid and detergent (MTAD), and EDTA with anionic detergent (EDTA-T) have been used to eliminate this adherent layer by demineralizing dentin; this, in turn, facilitates mechanical preparation of root canals and open dentin tubules for better penetration and diffusion of the antibacterial irrigation to act on the unreachable microbiota and its byproducts [3].

On the other hand, this decalcifying action might have a chemical and physical impact on dentin structure, affect the surface roughness and calcium/phosphorus ratio of dentin, decrease the microhardness of root canal dentin regardless of the duration or amount applied inside the canal, and cause possible alterations in dentin permeability and resin-bonding strength; consequently, coronal microleakage could take place with subsequent bacterial invasion [4]. Furthermore, the stability, durability, and adhesive properties of root canal filling materials might be weakened and, thus, the success rate of endodontic therapy could be affected in return [3,5,6]. Recently, BioAkt, a new chemical formula composed of silver ions 0.003% in citric acid 4.846% is currently under research to evaluate its benefits and drawbacks as an antibacterial chelating agent during endodontic therapy [7]. Interestingly, the microhardness of root dentin has been the main concern of recent studies while assessing the action of up-to-date chelating agents. Vickers hardness number (VHN) testing has been used for this purpose due to its accuracy and reliability in addition to the pyramidal indentation technique through which the mechanical characteristics of a very thin disc can be analysed [4,8,9]. 

Hence, this in vitro study aims to compare the impact of 17% EDTA, BioAkt, 10% citric acid, and 40% citric acid on the microhardness of root canal dentin.

## Materials and methods

Preparation of tooth specimens

**Figure 1:**
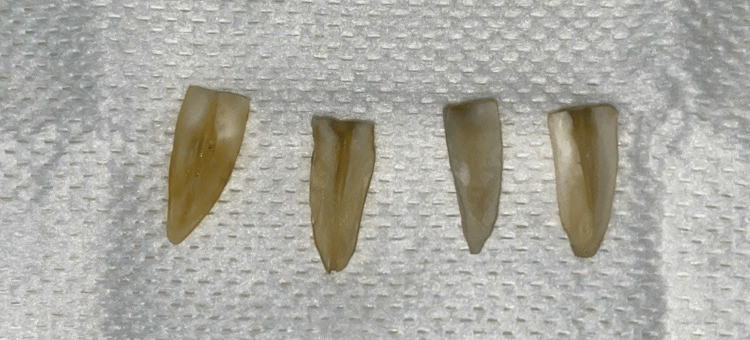
The roots after longitudinal splitting.

A total of 45 single-root teeth were collected from patients referred to the Faculty of Dentistry at Damascus University, Damascus, Syria, to extract teeth for orthodontic or periodontal treatment. Informed consent was obtained from patients before extraction. Immediately after extraction, the teeth were rinsed using running water, then immersed and disinfected in 2.5% NaOCL for one hour [8]. After that, the collected teeth were stored in a tightly closed plastic bottle, containing distilled water, inside a refrigerator at 4°Celsius, for no more than six months [10]. The teeth were examined under 2.5× magnifying loupes before the experiment to exclude any tooth with crack, fracture, caries, or enamel defect. The accepted 45 single-root teeth were decoronated using a diamond disc on a straight handpiece with cooling water. The aim of resection was to unify the sectioned roots with the same length of 16 millimeters (mm) [4]. After that, the root canals were probed using k-file size 10, then prepared to the working length (16 mm) using rotary files (ORODEKA Plex V, China) in the following sequence 20/0.4, 25/0.4, 25/0.6 with 5.25% NaOCL irrigation through 27-gauge side-vented needles. In the next step, the roots were split longitudinally, and the sound section was selected for the research (Figure 1).

The sectioned root was fixed over a resin block for better handling, and then the dentin surface was ground flat and smoothed using a series of ascending grades of carbide abrasive papers 500, 800, 1,000, and 1,200 grit (BIGO, Dent Product. Germany) with distilled water irrigation, and finally polished with 0.1 mm alumina suspension on a rotary felt disc (Microdont LDA. Brazil) to obtain a smooth glossy mirror-like surface [9]. Five solutions: distilled water, 17% EDTA, BioAkt, 40% citric acid, and 10% citric acid were selected for the laboratory study; the power of hydrogen PH was measured for each solution, using PH meter (OSK 14836 OGAWA Japan device), and the measured values were 6.7, 6, 1.9, 1.8, and 1.84 respectively.

Sampling distribution and pharmacological procedures

**Figure 2:**
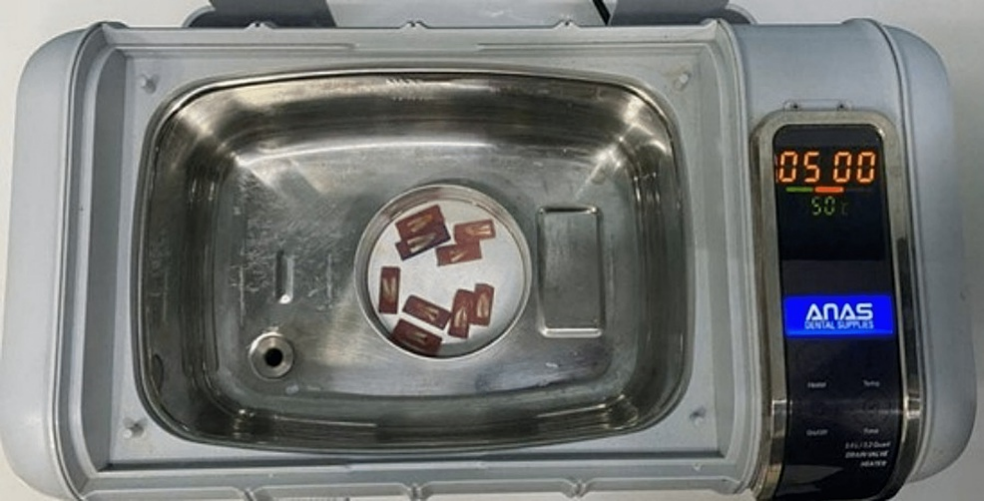
The samples inside the ultrasonic vibrator.

The 45 sectioned roots were randomly distributed, using the Research Randomizer program (Copyright © 1997-2022 by Geoffrey C. Urbaniak and Scott Plous), into five equal groups A, B, C, D and E, nine teeth each, treated with 40 millilitres (ml) of distilled water as a control, 17% EDTA, BioAkt, 40% citric acid, and 10% citric acid, respectively, inside an ultrasound vibrator for five minutes to ensure that the solution fully interacted with the root surface (Figure 2), then the treated roots were thoroughly rinsed with five ml of distilled water [9,11,12].

Microhardness measurement

**Figure 3:**
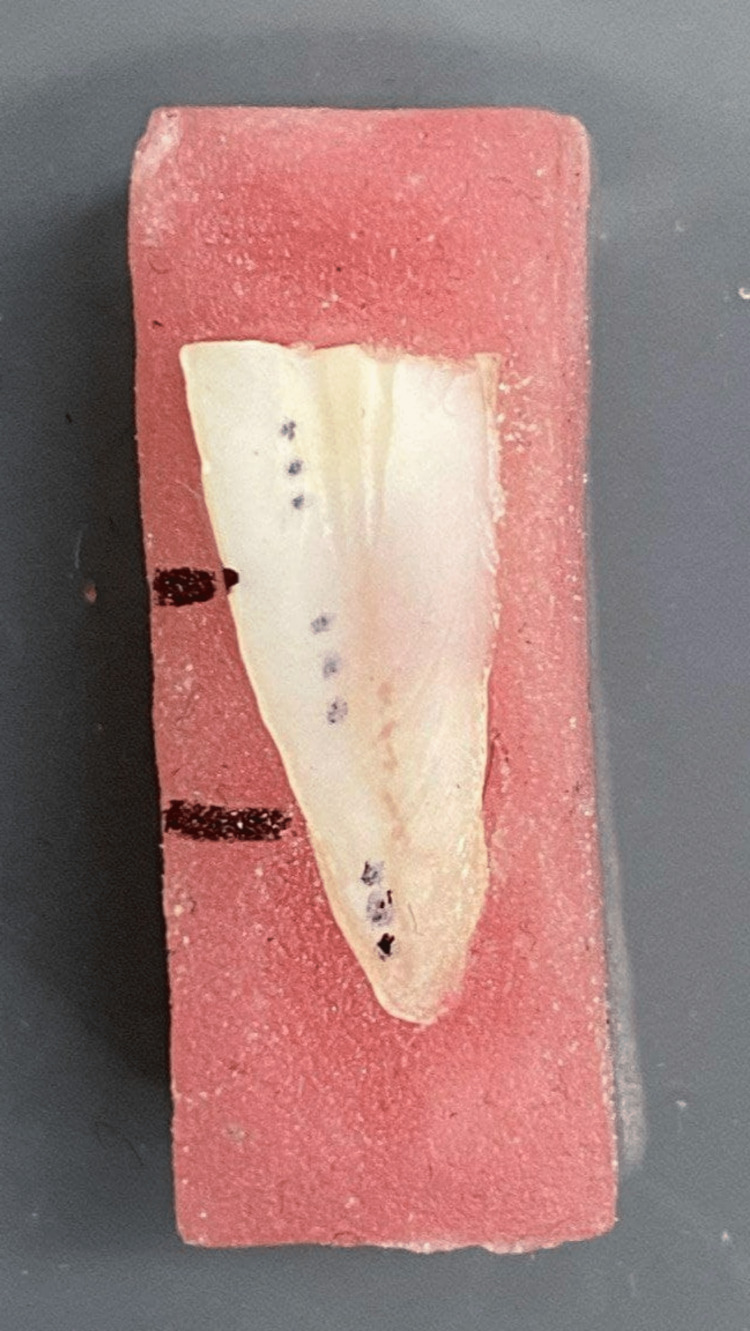
The root surface was marked close to canal borders.

The VHN of dentin was measured using a microhardness testing machine (Galileo Microscan OD, Italy) in the Faculty of Mechanical Engineering at Damascus University, according to the equation: HV=(2Psinθ/2)/D2HV=(2Psinθ/2)/D2 Where P means the applied load (Kgf), D represents the diagonal of indentation (mm), and θ stands for the angle between the opposite faces of diamond=136°. Each sample was fixed over the device base and 40X magnification with an appropriate illuminator was utilized to outline the root surface. Then, three points were marked on each of the coronal, medium, and apical parts of the root surface close to the canal borders (Figure 3).

**Figure 4:**
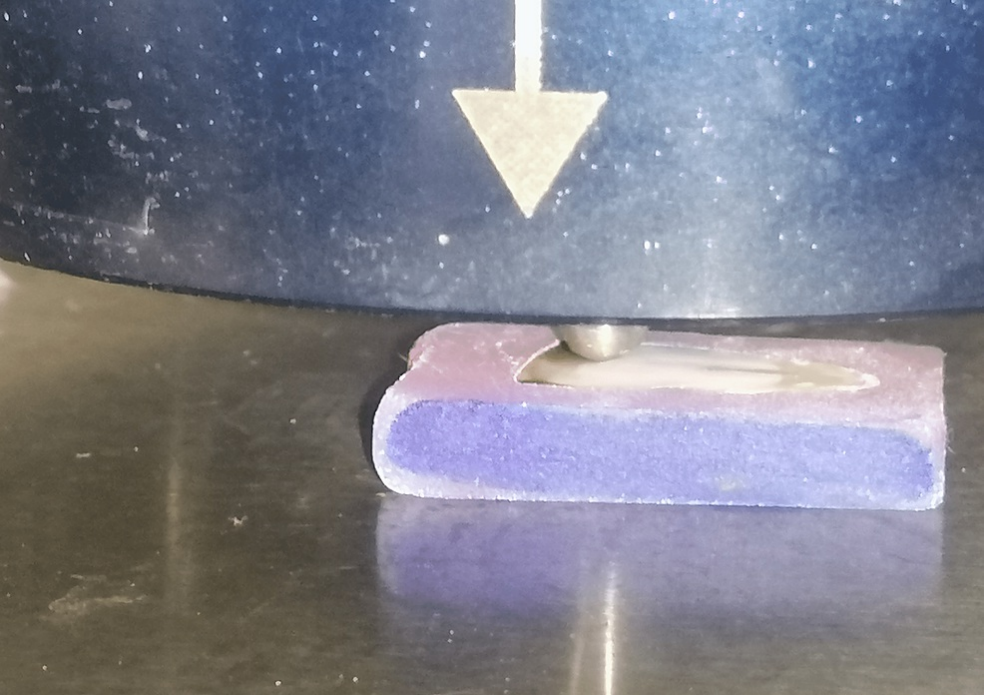
The prism on the specific point over the root surface.

**Figure 5:**
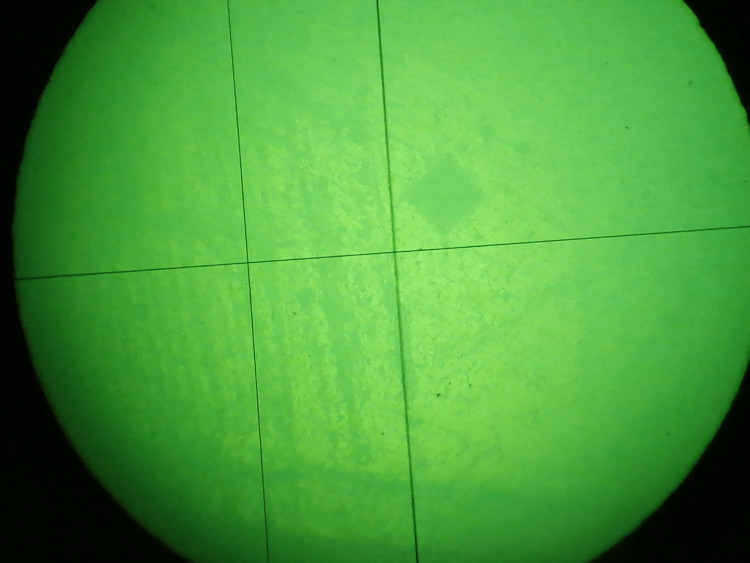
The rhomboid shape resulted from the impression of the prism on the root surface.

A diamond pyramid, 136-degree with 300 grams (g) weight, was directed and applied on the specific root surface for 15 seconds to form a rhomboid impression (Figure 4) [8]. The resulting rhomboid diagonal was utilized to calculate the microhardness electronically using the VHN (Figure 5). The arithmetic average was calculated for each root part separately and for the whole root in total [13]. Microhardness measurements were collected before and after treating the sample with the specific solution.

Statistical analysis

IBM SPSS Statistics for Windows, Version 24.0 (Released 2016; IBM Corp., Armonk, New York, United States) was utilized for analysing data using paired sample T-test, one-way ANOVA test, and least significant difference (LSD) test (multiple comparisons) (P < 0.05).

## Results

**Figure 6:**
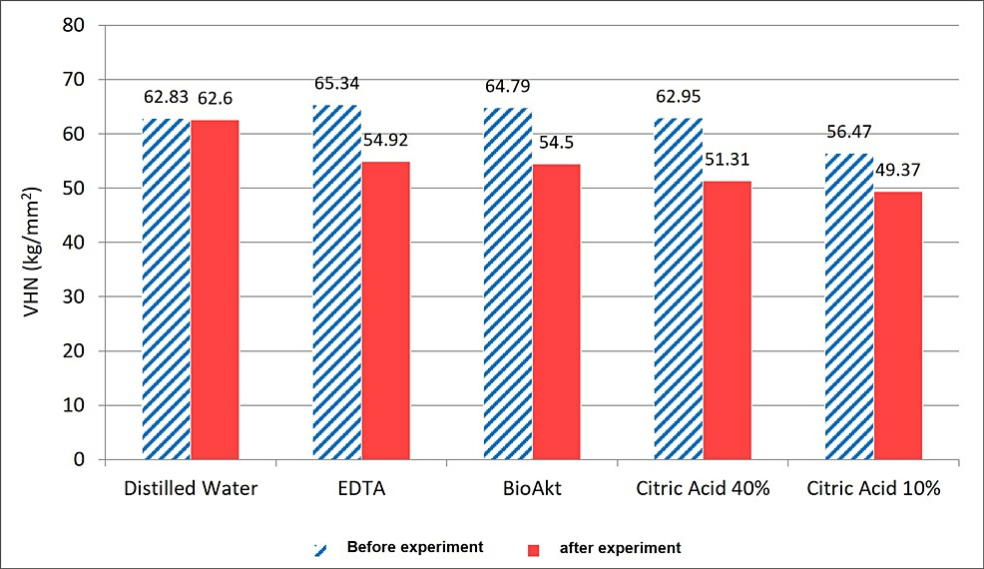
The arithmetic averages of dentin microhardness (VHN) before and after the experiment in the five groups. EDTA: ethylenediamine tetraacetic acid; VHN: Vickers hardness number

The arithmetic averages of dentin microhardness values before the experiment were 62.83kg/mm², 65.34 kg/mm², 64.79 kg/mm², 62.95 kg/mm², and 56.47 kg/mm² for the groups of distilled water, 17% EDTA, BioAkt, 40% citric acid, and 10% citric acid, respectively, while the averages after the experiment were 62.60 kg/mm², 54.92 kg/mm², 54.50 kg/mm², 51.31 kg/mm², and 49.37kg/mm² for groups A, B, C, D, and E, accordingly (Figure 6). Except for the control group A, the microhardness differences before and after the experiment in groups B, C, D, and E were statistically significant using paired sample T-test (Table 1).

**Table 1:** The statistical differences in dentin microhardness (VHN) using paired sample T-test before and after the experiment in each group. EDTA: ethylenediamine tetraacetic acid; VHN: Vickers hardness number

Group		Averages of VHN (kg/mm²)	SD	t-test	p-value
Distilled Water	before	62.83	6.730	0.074	0.942
	after	62.60	6.645		
EDTA	before	65.34	6.621	7.374	0.000078
	after	54.92	6.965		
BioAkt	before	64.79	6.003	4.279	0.003
	after	54.50	5.946		
Citric Acid 40%	before	62.95	5.716	4.781	0.001
	after	51.31	6.097		
Citric Acid 10%	before	56.47	3.876	10.931	0.000004
	after	49.37	3.891		

One-way ANOVA test revealed that there were significant differences among the five groups regarding the microhardness of root dentin after the experiment (Table 2). In addition, the LSD test, multiple comparisons, showed that the averages of dentin microhardness after the experiment in groups B, C, D, and E were significantly lower than the control group A, without statistical differences among the former groups B, C, D, and E (Table 3).

**Table 2:** Results of one-way ANOVA analysis of dentin microhardness (VHN) after the experiment in the five groups. EDTA: ethylenediamine tetraacetic acid; VHN: Vickers hardness number

The average of VHN (kg/mm²)	Distilled Water	17% EDTA	BioAkt	40% Citric Acid	10% Citric Acid	F	p-value
Min –max	73.17-53.13	61.9-48	64.67-46.6	60.33-40.07	56.1-44.43	7.568	0.000123
Mean ± SD.	6.65 ± 62.6	4.48 ± 54.92	5.95 ± 54.5	6.1 ± 51.31	3.89 ± 49.37		
Median	61.47	53.5	64.67	53.4	49.37		

**Table 3:** The results of LSD test (multiple comparisons) for dentin microhardness (VHN) after the experiment in the five groups. * P < 0.05: there is a statistical difference between the two averages. EDTA: ethylenediamine tetraacetic acid; VHN: Vickers hardness number; LSD: least significant difference

Group		Difference between each two averages of VHN (kg/mm²)	p-value
17% EDTA	distilled water	10.19	0.000111^*^
	BioAkt	0.14	0.954
	40% Citric acid	-1.22	0.611
	10% Citric acid	3.32	0.170
BioAkt	distilled water	10.05	0.000133^*^
	40% Citric acid	-1.36	0.572
	10% Citric acid	3.19	0.188
40% Citric acid	distilled water	11.41	0.000022^*^
	10% Citric acid	4.54	0.063
10% Citric acid	distilled water	6.87	0.006^*^

## Discussion

Successful irrigation of the root canal system is considered a mandatory step for the healing process and long-term results of endodontic therapy. Different types of solutions have been used to disinfect the root canals before obturation [3,14]. NaOCL is an important irrigant characterized by bactericidal, lubricant, and organic solvent properties, but is considered ineffective against the smear layer. Therefore, it has been used alternately with chelating agents such as EDTA to remove this adherent layer of debris by affecting the inorganic mineral components of dentin [2,3]. This, in turn, will negatively decrease the microhardness of root dentin [5,6]. Research has studied different concentrations of citric acid ranging from 1% to 50% to look for a substitute for EDTA with a lesser impact on dentin [15,16]. Actually, the purpose of selecting 40% citric acid in this study, which is four times more concentrated than 10%, is to detect the effect of a higher concentration on the microhardness of root canal dentin, while the reason for comparing the chelating solutions with EDTA is the well-researched and traditional use of the latter. BioAkt is an innovative endodontic irrigant consisting of citric acid (4.846%) and silver electrolytes (0.003%) in addition to detergents and water [7]. The chelating effect of BioAkt is due to the presence of citric acid [17], while the bactericidal characteristic is mainly contributed to silver ions; this unique composition specifies the dual function of BioAkt during endodontic treatment [18,19]. Therefore, this novel chelating agent was chosen in this study to assess its effect on dentin microhardness.

Many studies utilized the microhardness test to assess the effects of chelating agents on dentin structure after endodontic treatment [19-21]. Interestingly, the four chelating solutions 17% EDTA, BioAkt, 40% citric acid, and 10% citric acid in this laboratory study caused the same significant decline in dentin microhardness after five minutes of application. This result is compatible with the study of Cruz-Filho et al. [6], Ballal et al. [22], and Scelza et al. [2]. However, the results of this study are different from the Eldeniz et al. study, where 10% citric acid reduced dentin microhardness more than 17% EDTA after 90 seconds of application [23]. Indeed, research revealed that the crucial factor controlling the efficacy of chelating agents is depending mainly on PH value regardless of its concentration [24-28]. Therefore, 17% EDTA has less ability to chelate with calcium ions in dentin due to its neutral PH [25]. Furthermore, 5% citric acid could remove the smear layer at PH=1.9 but turned out to be ineffective at PH=6 [24]. Actually, the three chelating solutions BioAkt, 40% citric acid, and 10% citric acid were the same in affecting dentin microhardness in this study, because they already had similar PH. Interestingly, 17% EDTA had a noticeably higher PH but decreased the microhardness similarly to them; this can be explained by the use of ultrasonic waves during the application of the chelating solution, which probably activated 17% EDTA to easily affect and interact with root canal dentin [28,29].

As a matter of fact, this laboratory study has many limitations including the way of applying the chelating solution over the whole surface of the sectioned root compared with the restricted irrigation inside the root canal system clinically in addition to the pressure and heat possibly generated during the laboratory preparation stages of the tooth specimen including root canal treatment, resection, splitting, grinding and smoothing, which might add an additional impact on dentin microhardness. Finally, only one duration, five minutes of treatment with the specific solution, was considered to compare the chelating solutions in this study, which might not represent the irregular application periods inside the root canal clinically.

## Conclusions

Within the limitations of this laboratory study, the four chelating agents 17% EDTA, BioAkt, 10% citric acid, and 40% citric acid caused the same significant decline in the microhardness of root canal dentin. This result might have possible reflections on the properties of root canal filling materials and endodontic therapy as well.
